# Soil Abiotic Properties and Plant Functional Traits Mediate Associations Between Soil Microbial and Plant Communities During a Secondary Forest Succession on the Loess Plateau

**DOI:** 10.3389/fmicb.2019.00895

**Published:** 2019-04-26

**Authors:** Yongfu Chai, Ying Cao, Ming Yue, Tingting Tian, Qiulong Yin, Han Dang, Jiaxin Quan, Ruichang Zhang, Mao Wang

**Affiliations:** ^1^Key Laboratory of Resource Biology and Biotechnology in Western China, Ministry of Education, Northwest University, Xi’an, China; ^2^School of Life Sciences, Northwest University, Xi’an, China; ^3^Department of Plant Ecology, University of Tübingen, Tübingen, Germany; ^4^College of Grassland and Environment Sciences, Xinjiang Agricultural University, Ürümqi, China

**Keywords:** forest succession, aboveground–belowground interactions, bacterial community, fungal community, soil properties, plant traits

## Abstract

In the context of secondary forest succession, aboveground-belowground interactions are known to affect the dynamics and functional structure of plant communities. However, the links between soil microbial communities, soil abiotic properties, plant functional traits in the case of semi-arid and arid ecosystems, are unclear. In this study, we investigated the changes in soil microbial species diversity and community composition, and the corresponding effects of soil abiotic properties and plant functional traits, during a ≥150-year secondary forest succession on the Loess Plateau, which represents a typical semi-arid ecosystem in China. Plant community fragments were assigned to six successional stages: 1–4, 4–8, 8–15, 15–50, 50–100, and 100–150 years after abandonment. Bacterial and fungal communities were analyzed by high-throughput sequencing of the V4 hypervariable region of the 16S rRNA gene and the internal transcribed spacer (ITS2) region of the rRNA operon, respectively. A multivariate variation-partitioning approach was used to estimate the contributions of soil properties and plant traits to the observed microbial community composition. We found considerable differences in bacterial and fungal community compositions between the early (S1–S3) and later (S4–S6) successional stages. In total, 18 and 12 unique families were, respectively, obtained for bacteria and fungi, as indicators of microbial community succession across the six stages. Bacterial alpha diversity was positively correlated with plant species alpha diversity, while fungal diversity was negatively correlated with plant species diversity. Certain fungal and bacterial taxa appeared to be associated with the occurrence of dominant plant species at different successional stages. Soil properties (pH, total N, total C, NH_4_-N, NO_3_-N, and PO_4_-P concentrations) and plant traits explained 63.80% and 56.68% of total variance in bacterial and fungal community compositions, respectively. These results indicate that soil microbial communities are coupled with plant communities via the mediation of microbial species diversity and community composition over a long-term secondary forest succession in the semi-arid ecosystem. The bacterial and fungal communities show distinct patterns in response to plant community succession, according to both soil abiotic properties and plant functional traits.

## Introduction

Continued deforestation has resulted in the expansion of secondary forest areas in terrestrial ecosystems. Therefore, it is becoming increasingly important to understand the successional processes of secondary forests (Bruelheide et al., 2011). Forest community succession is driven by aboveground–belowground interactions (Bardgett et al., 2005; Kardol et al., 2006). As aboveground members of the forest community, the general patterns of plant community composition along successional gradients are well known for many forest types (e.g., Howard and Lee, 2003; Matthews et al., 2009). Since soil microbes play a key role in plant species coexistence and community structure, they are increasingly being recognized as belowground drivers of plant diversity in terrestrial ecosystems (van der Heijden et al., 2008; Klironomos et al., 2011; Delgado-Baquerizo et al., 2018). In addition, soil microbes generally have rapid responses and high turnover rates in the face of alterations in the environment; thus, soil microbes may provide additional information about the succession mechanism and an early indication of the restoration trajectory after ecosystem disturbances (Banning et al., 2011).

For a given secondary succession series, there is generally a predictable species turnover as the characteristic species composition emerges at different time points with the development of the system (Bruelheide et al., 2011; Måren et al., 2017). The characteristic plant species composition at different successional stages has varying effects on the soil environment, by altering the abiotic properties and influencing the microbial community composition (Zhang et al., 2016; Yeoh et al., 2017). Both bacteria and fungi are major components of the soil microbiota; they may perform diverse ecological functions due to differences in their life history, phenotype, and phylogeny (Sun S. et al., 2017). Some studies have reported the community composition patterns of soil bacteria or fungi in relation to the secondary grassland succession in sandy lands (Zuo et al., 2016), the Loess Plateau region (Li et al., 2013; Xiao et al., 2016; Zhang et al., 2016), chalk grasslands (Kuramae et al., 2010), and temperate upland grasslands (Millard and Singh, 2010). However, inconsistent relationships were found between the plant community and the soil microbial community in these grasslands because of heterogeneity in the vegetation, climatic conditions, and physical landforms across the gradients. This inconsistency existed even among other forest successional series, according to a few studies that investigated secondary forest succession after mining (Banning et al., 2011; Zumsteg et al., 2012; Sun S. et al., 2017) or glacial retreat (Jangid et al., 2013). It was also reported that soil bacteria exhibited faster recovery than the fungal community during forest succession after mining (Banning et al., 2011). So far, there are still gaps in our understanding of secondary forest succession in arid and semi-arid regions where plant species composition and diversity usually undergo changes quite rapidly (Lozano et al., 2014).

Soil abiotic properties, for example, pH and nutrient availability, are key regulators that link plant performance with soil microbial communities (Bannert et al., 2011; Prescott and Grayston, 2013). Plant community succession occurs when early plant-induced changes in soil nutrient availability improve the growth and establishment of future plants (Clements, 1916; van der Putten et al., 2013). Therefore, while soil conditions could affect the environmental filtering of plant functional traits during community succession (McGill et al., 2006), plant traits may also, in turn, influence community dynamics through their effects on soil microbial communities (Demenois et al., 2018). In fact, growing evidence demonstrates that plant traits may be important drivers of the inter-relationships between plants versus soil abiotic properties and microbial communities (Grigulis et al., 2013; Legay et al., 2014; Cantarel et al., 2015; Ke et al., 2015; Moreau et al., 2015). At the species level, variation in soil microbial communities alters the environmentally driven selection pattern of plant traits, such as root traits (Orwin et al., 2010; Pohl et al., 2011; Birouste et al., 2012) and leaf traits (Laughlin, 2011). At the community level, community-weighted mean (CWM) plant traits, such as leaf nitrogen concentration (LNC) and specific leaf area (SLA), can improve our understanding of soil microbial community diversity and composition at a larger spatial scale (e.g., de Vries et al., 2012). During plant community succession, it is generally accepted that nutrient-poor early successional communities are dominated by fast-growing exploitative species that feature high SLA, LNC, and root nitrogen concentration (RNC) (Navas et al., 2010; Chai et al., 2016). Conversely, nutrient-rich later successional communities are dominated by slow-growing conservative species that feature the opposite traits (Navas et al., 2010). Despite these known patterns, none of these studies have quantitatively assessed the relative contributions of soil abiotic properties and plant functional traits to the soil microbial community characteristics in relation to secondary forest succession.

The Loess Plateau in northern China is a semi-arid ecosystem that is well known for its deep loess and severe soil water depletion (Wang et al., 2008). The majority of the plant communities on this plateau are currently in the successional transition from abandoned agricultural land to a forest climax community that reflects anthropogenic disturbances at different time points since the 1860s (Zhu, 1993; Chai et al., 2016). A long-term forest succession beginning with an abandoned land represents an opportunity to study the entire secondary forest succession under similar starting conditions. Here, we determined the associations between soil bacterial and fungal community characteristics versus plant community diversity and functional traits over a long-term (about 150 years) arable-to-climax forest succession on the Loess Plateau. The aim of this study was (1) to investigate the patterns of change in the composition and diversity of soil bacterial and fungal communities; (2) to determine whether there are consistent patterns in the coupling of soil microbial and plant communities, and whether characteristic microbial groups would emerge at different successional stages of the plant community; and (3) to determine how the soil microbial community characteristics are related to soil abiotic properties and plant functional traits during secondary forest succession on the Loess Plateau.

## Materials and Methods

### Study Site

**FIGURE 1:**
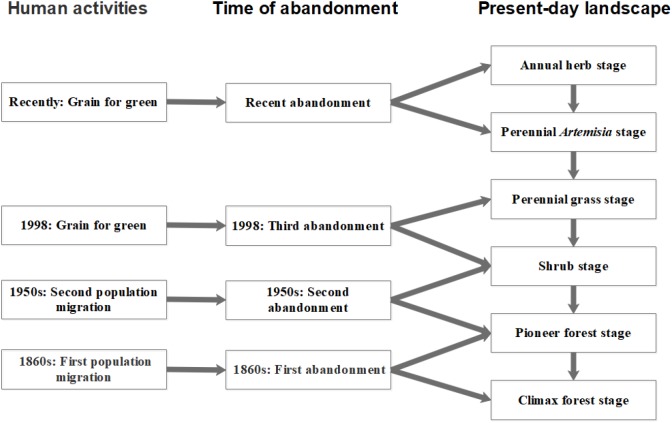
A successional model of the plant community following the abandonment of agricultural fields on the Loess Plateau of China.

The study site was located in the Ziwuling reserve region (35°09′ to 35°40′ N, 108°47′ to 108°57′ E) of the Loess Plateau, Shaanxi, China. This region has a semi-arid, temperate, continental monsoon climate (Zhu, 1993). The mean annual precipitation ranges from 550 to 650 mm, and it is mostly concentrated in summer. The mean annual temperature is 9–11°C, and the elevation is 1,100–1,150 m. Population migration and the grain-for-green policy have led to different levels of restoration of the natural vegetation since the 1860s (Zhu, 1993) (Figure 1), and the study site (which covers an area of 12 × 15 km) comprises a complex vegetation mixture of forest, shrub, and meadow. Large areas of these communities are now at different stages of forest succession (Zhu, 1993). All community fragments in the present-day landscape were classified according to their successional age with the help of local chronicles and previous surveys (Zhu, 1993; Chai et al., 2016). The communities were assigned to one of six successional stages (S1–S6), namely 1–4, 4–8, 8–15, 15–50, 50–100, and 100–150 years after abandonment. Generally, stage 1 was dominated by annuals, while stage 2 was dominated by perennials (e.g., *Artemisia gmelinii* and *Artemisia lavandulaefolia* [Compositae]). Stage 3 and stage 4 were dominated by perennial grass (Gramineae) and shrub, respectively. At stage 5, pioneer tree species became the dominant growth form. At stage 6 (about 150 years), species of the genus *Quercus* became dominant (Supplementary Table S1).

Using the method of substitution of space for time (Walker et al., 2013), 30 permanently marked plots (five plots per stage) were established between June and September 2013, to investigate the vegetation dynamics following abandonment. A distance of more than 1 km was maintained between any two plots in order to include different community types and avoid spatial autocorrelation and pseudo-replications (Kinupp and Magnusson, 2005). The size of each plot was 20 m × 20 m. The selection of plots was mainly limited by the lack of reliable knowledge on land-use history. Nonetheless, we established 30 plots that we believe were subjected to the same agricultural history and are presently on a similar trajectory toward becoming a climax forest under natural conditions. The plots were surveyed in 2014. All vascular species within each plot were identified, and the abundance, coverage, height, and life form (woody vs. herbaceous) of the species were documented. Pre-analyses showed that there was only small variation between the plots in terms of elevation (1,000–1,040 m) and slope (18°–25°), so these factors were not considered in the later analyses.

### Plant Sampling and Trait Measurements

In total, we measured 15 plant functional traits (Supplementary Table S2) of 210 species that were potentially related to plant–soil interactions (Philippot et al., 2013). These traits were chosen to represent the multi-organ functions of plants associated with resource use, competitive ability, and successional strategy. All traits were measured for five or more individuals per species within each plot randomly, and the measurements generally followed the protocols of Cornelissen et al. (2003). Rare species (number of individuals < 5) were not sampled.

Vegetative plant height was measured as the distance between the top of the photosynthetic tissues of each individual and the soil surface, at the end of the growing season. For the woody species, completely developed sun-exposed leaves were sampled randomly from five healthy branches (10-20 leaves per individual). The stem and root of the same individual were also sampled. Roots were sampled at 20–30 cm of soil depth near the plant basal stem. For the herbaceous species, leaves, stems, and roots were sampled from five random 0.5 × 0.5 m quadrants within each plot.

The fresh mass (*M*_w_) of each leaf, stem, and root sample was measured immediately with an SE202F electronic balance (Ohaus Corp., Parsippany, NJ, United States). The leaf surface area of most species was calculated using the Motic Images Plus 2.0 (Motic China Group Co., Ltd., Xiamen, China) software. In particular, the leaf surface area of *Pinus tabuliformis* was estimated over a cylindrical area, according to the method of Yan et al. (2006). The dry mass (*M*_D_) of all the samples was determined after 72 h of drying at 80°C in a drying oven. The SLA was measured as the ratio of leaf surface area to dry mass (cm^2^⋅g^-1^). The dry matter content of the leaves (LDMC), stems (SDMC), and roots (RDMC) was calculated as *M*_D_/*M*_w_. The stem specific density (SSD) was calculated as the stem dry mass divided by its fresh volume (Riva et al., 2016).

The carbon and nitrogen concentrations of the leaves (LCC and LNC), stems (SCC and SNC), and roots (RCC and RNC) were determined with an elemental analyzer (Euro Vector EA3000, Milan, Italy). The total leaf phosphorus concentration was determined using the ammonium molybdate spectrophotometric method following H_2_SO_4_-H_2_O_2_ digestion (Bowman, 1988).

Seed mass (SM) data (dry mass per 1,000 seeds [mg]) were obtained through field collections for the majority of the species. Since SM is considered to be less plastic (Violle et al., 2009), we used the species mean value as the best estimate.

### Soil Sampling and Abiotic Analysis

Soil samples were collected using a 5-cm (diameter) stainless steel corer from the top 20 cm of the soil profiles after the litter horizons were removed. A total of 12 soil cores were collected for each plot along an S-shaped pattern and mixed into one composite sample. Each soil core was obtained within a radius of 0.75 m from an area that was away from lichens, biological crusts, and any other vegetation. Each soil sample was divided into two subsamples after removing visible plant roots, stones, and litter. One subsample was immediately stored at -80°C for DNA analysis, and the other subsample was air-dried for analysis of abiotic properties. Soil pH was measured in a 1:2.5 (w/w) soil: water suspension with a pH electrode (PHS-3C; Shanghai REX Instrument Factory, Shanghai, China). In addition, ammonium nitrogen (NH_4_-N), nitrite nitrogen (NO_3_-N), and phosphate phosphorus (PO_4_-P) were analyzed using a high-performance micro flow analyzer (QuAAtro; SEAL Analytical GmbH, Norderstedt, Germany). Soil total carbon and nitrogen concentrations were determined with an elemental analyzer (Euro Vector EA3000, Milan, Italy).

### Sequencing Analysis

Total genomic DNA was extracted from 0.4 g of soil samples using the MO BIO Soil Isolation Kit (MO BIO, Carlsbad, CA, United States) according to the manufacturer’s instructions. Sequencing analysis of soil bacterial and fungal communities was performed by targeting the V4 hypervariable region of the 16S rRNA gene (Carini et al., 2016) and the internal transcribed spacer (ITS2) region of the rRNA operon (Yang et al., 2017), respectively, on an Illumina Miseq PE250 platform (Illumina Inc., San Diego, CA, United States) at Novogene Cooperation (Beijing, China). Raw pair-end reads were quality filtered using the QIIME pipeline (Caporaso et al., 2010). Chimeric sequences were removed using the USEARCH software with the UCHIME algorithm (Edgar et al., 2011). The remaining sequences were assigned to operational taxonomic units (OTUs) at a threshold of 97% similarity using the UCLUST model (Edgar, 2010). Representative sequences for each OTU were assigned to taxonomic groups using the RDP classifier at a threshold of 80% (Wang et al., 2007).

### Statistical Analyses

All statistical analyses were conducted with the R software (version 3.1.2; R Development Core Team, 2014). Based on the UniFrac distances, we performed principal coordinate analysis (PCoA) to assess the differences in microbial community composition between soil samples from different successional stages, by using the “pcoa” function of the “vegan” package (Oksanen et al., 2016). The Adonis and Anosim statistical tests were then performed to verify whether there were significant differences between successional stages. Hierarchical clustering analysis was used to assess the differences in abundance distribution of the top 40 dominant genera across the six successional stages. Heatmaps were then generated using custom R scripts. Indicator values (IndVals) were used to predict the microbial communities associated with specific successional stages. Microbial indicators at the family level were estimated by using the “indval” function of the “labdsv” package.

We calculated the CWM value of plant traits (*P_j_* in Eq. 1) by using trait-gradient analysis (Ackerly and Cornwell, 2007), as shown below.

$$P_{j}=\frac{\Sigma_{i=1}^{S}a_{ij}\times t_{ij}}{\Sigma_{i=1}^{S}a_{ij}}$$

where *t_ij_* and *a_ij_* represent the trait value and relative coverage of species *i* in plot *j*, respectively, and *S* and *P* represent the total number of species and plots, respectively.

Plant and microbial alpha diversity were characterized by the Shannon and Simpson diversity indices (Koleff et al., 2003). The Chao1 index of bacterial and fungal communities was calculated and used as an indicator of microbial species richness (Caporaso et al., 2010). The Cody index was used as an indicator of the turnover rate of both microbial and plant species between successional stages (Magurran, 1988). The differences in plant traits and species diversity, as well as those in soil properties and microbial diversity across the six successional stages were examined by one-way analysis of variance (ANOVA). Relationships between the soil properties, plant community diversity and microbial community diversity were assessed using principal component analysis (PCA) with the “vegan” and “ggbiplot” packages.

The important value index of plant species was calculated as described by Gautam et al. (2018) and its association with the abundance of the dominant microbial groups was determined by a classical canonical correspondence analysis (CCA). Classical CCA was also used to investigate the effects of soil properties and plant traits on bacterial and fungal community compositions, while the Monte Carlo permutation was used to examine the significance of these effects. Microbial community data was composed by relative abundance of 62,501 OTUs for bacteria and 5,662 OTUs for fungi after removing singleton in 30 samples. A step function was used to choose the plant traits before CCA to minimize the Akaike information criterion. The *envfit* function of the “vegan” package was used as described previously (Wietz et al., 2015), to determine which soil abiotic properties and plant functional traits were significant in explaining the soil microbial data. We also used a multivariate variation-partitioning approach (Borcard et al., 1992) to decompose the total variation of the microbial community composition into (1) variation solely explained by the plant traits, (2) variation solely explained by the soil properties, (3) variation explained by both the soil properties and plant traits, and (4) unexplained variation.

Co-occurrence networks can provide a visual analysis of ecological information and have the potential to clarify complex microbial community structures (Barberán et al., 2012). Herein, co-occurrence network analysis was used to explore the co-occurrence patterns of microbial taxa (genera in this study) and the biotic interactions between bacteria and fungi. Topological analysis of networks was performed in terms of degree, path length, clustering coefficient (the degree to which nodes tend to cluster together), and modularity (>0.4 is considered to indicate an evident modular structure) (Sun W. et al., 2017).

## Results

### Soil Microbial Community Diversity and Composition Across the Six Successional Stages

**FIGURE 2:**
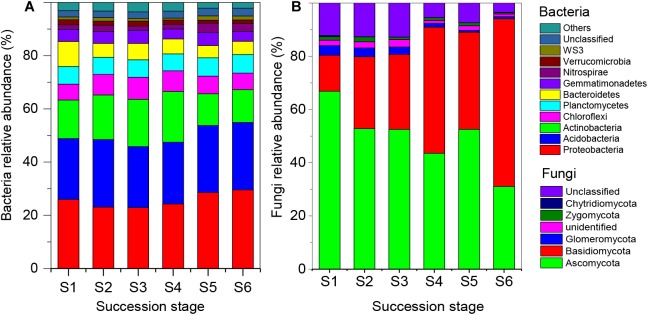
Relative abundance of soil bacterial **(A)** and fungal **(B)** communities at the phylum level across the six successional stages (S1–S6 represent 1–4, 4–8, 8–15, 15–50, 50–100, and 100–150 years after abandonment, respectively). The abundance of each taxon in relation to the abundance of all the taxa was calculated as the relative abundance (based on the data of sequence analysis).

After quality trimming, a total of 1,743,939 bacterial and 1,935,570 fungal high-quality sequences remained, from which 62,501 and 5,662 OTUs were identified, respectively. At the phylum level, the soil bacterial communities were dominated by Proteobacteria (25.7% of total bacterial sequences), Acidobacteria (24.2%), Actinobacteria (15.4%), Chloroflexi (7.1%), Planctomycetes (6.6%), and Bacteroidetes (6.0%). The relative abundance of these dominant bacterial phyla did not differ significantly across the six successional stages (Figure 2A). The fungal communities were dominated by Ascomycota (49.9% of total fungal sequences) and Basidiomycota (36.0%) across the six successional stages (Figure 2B). The relative abundance of Ascomycota increased along the successional gradient, while the abundance of Basidiomycota decreased (Supplementary Figure S1). The three alpha diversity indices (Simpson, Shannon, and Chao1) revealed similar patterns: that is, bacterial diversity increased along the successional gradient, while fungal diversity increased first and then declined (Supplementary Table S3).

**FIGURE 3:**
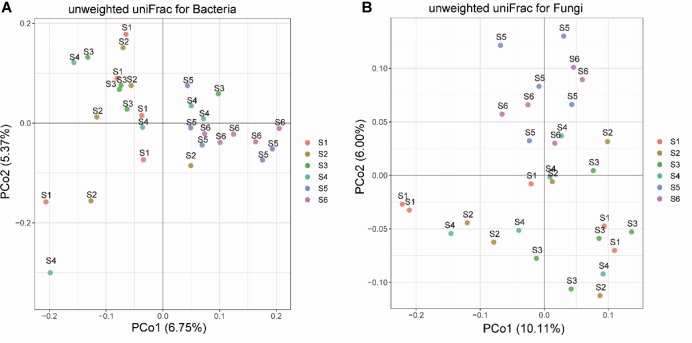
Principal coordinate analysis (PCoA) of soil bacterial **(A)** and fungal **(B)** community dissimilarities based on Unifrac distances among samples from the six successional stages (S1–S6 represent 1–4, 4–8, 8–15, 15–50, 50–100, and 100–150 years after abandonment, respectively).

Principal coordinate analysis analyses showed that the soil bacterial and fungal communities of different successional stages were distinct, as the communities of earlier stages (I-III) clustered together and those of later stages (IV-VI) clustered together separately (Figure 3). Both the Adonis and Anosim tests confirmed that plant community succession had a significant effect on microbial community composition (Supplementary Table S4). Hierarchical clustering of the data of the dominant genera provided consistent results: that is, microbial communities predominantly clustered according to successional stages (Supplementary Figures S2, S3), and the composition of early communities was distinct from that of later communities. Moreover, hierarchical clustering analyses revealed that certain bacterial and fungal genera were dominant at different successional stages. InVals analyses at the family level showed that each successional stage, with the exception of stage 2 had its unique indicator families for either bacteria or fungi. In total, 18 and 12 families were selected as indicators for bacterial and fungal communities, respectively, across the six successional stages (Table 1).

**Table 1:** Indicator families for soil bacterial and fungal communities across the six successional stages during a secondary forest succession on the Loess Plateau.

Successional stage	Vegetation type	Indicator family (bacteria)	Indicator value	*P*
S1	Annual herb	Nitrosomonadaceae	0.39	0.05
		Cytophagaceae	0.30	0.01
S2	Perennial Artemisia	–	–	–
S3	Perennial grass	Aurantimonadaceae	0.56	0.01
		Williamsiaceae	0.45	0.01
		Verrucomicrobiaceae	0.36	0.02
		Intrasporangiaceae	0.31	0.03
		Caldilineaceae	0.26	0.01
S4	Shrub	Tsukamurellaceae	0.48	0.04
		Streptomycetaceae	0.30	0.03
		Pseudonocardiaceae	0.28	0.04
S5	Pioneer forest	Alcaligenaceae	0.30	0.05
		Syntrophobacteraceae	0.27	0.01
		Entotheonellaceae	0.27	0.03
S6	Climax forest	Acidobacteriaceae	0.63	0.01
		Hyphomonadaceae	0.44	0.03
		Leptospirillaceae	0.44	0.01
		Hyphomicrobiaceae	0.27	0.01
		Planctomycetaceae	0.24	0.04
		**Indicator family (fungi)**		
S1	Annual herb	Spizellomycetaceae	0.62	0.04
S2	Perennial Artemisia	Septobasidiaceae	0.66	0.00
S3	Perennial grass	Halosphaeriaceae	0.91	0.05
		Leptosphaeriaceae	0.75	0.01
		Chaetothyriaceae	0.63	0.00
		Montagnulaceae	0.61	0.01
		Ceratobasidiaceae	0.50	0.04
		Tremellaceae	0.44	0.01
		Mycosphaerellaceae	0.42	0.03
S4	Shrub	Thelephoraceae	0.54	0.02
S5	Pioneer forest	Mycenaceae	0.61	0.03
S6	Climax forest	Stictidaceae	0.68	0.02


### Effects of Soil Abiotic Properties and Plant Functional Traits on Soil Microbial Community Composition

The soil abiotic properties differed significantly among the six successional stages (Supplementary Figure S4). Long-term community succession led to a considerable increase in soil total N, total C, NH_4_-N and PO_4_-P concentrations and a decline in soil NO_3_-N concentration and pH level. The later successional plant communities exhibited higher RCC, SCC, LCC, RDMC, SDMC, and LDMC than the early successional communities. In contrast, SLA, SNC, and LPC were significantly higher for the community at the youngest stage and lower for the community at the oldest stage (Supplementary Figures S5–S8). Changes in the CWM plant traits during succession represented a change in plant functional strategies from exploitative to conservative ones.

**FIGURE 4:**
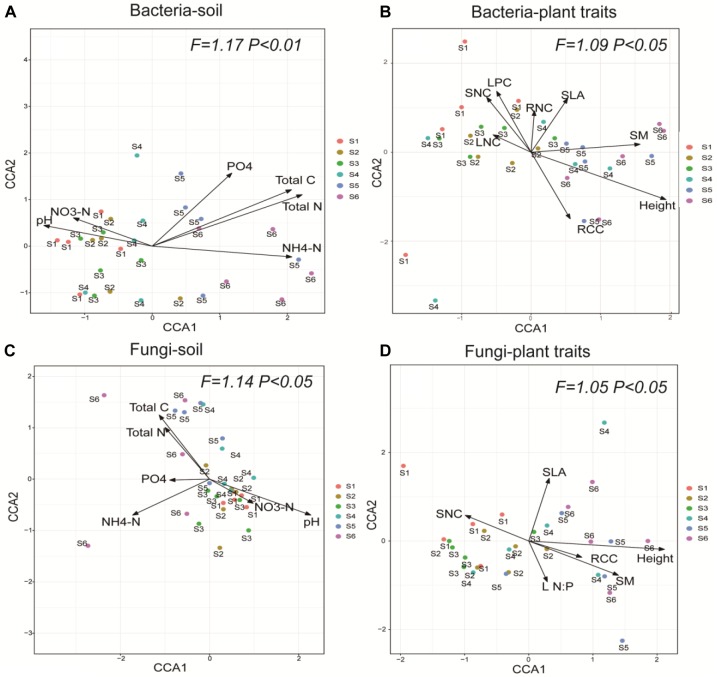
Canonical correspondence analysis showing the effects of soil abiotic properties and plant functional traits on the community composition of soil bacteria **(A,B)** and fungi **(C,D)** across the six successional stages (S1–S6 represent 1–4, 4–8, 8–15, 15–50, 50–100, and 100–150 years after abandonment, respectively). Soil properties: pH, total carbon (Total C), total nitrogen (Total N), available phosphorus (PO_4_-P), ammonium nitrogen (NH_4_-N), and nitrate nitrogen (NO_3_-N). Plant traits: RCC, root carbon content; LNC, leaf nitrogen content; SNC, stem nitrogen content; RNC, root nitrogen content; LPC, leaf phosphorus content; SM, seed mass; SLA, specific leaf area; L N:P, leaf N:P ratio; Height, plant height. *F*-values of Monte Carlo permutation testing for the effect of soil properties and plant traits are provided, with the level of significance set at *P* < 0.05.

**FIGURE 5:**
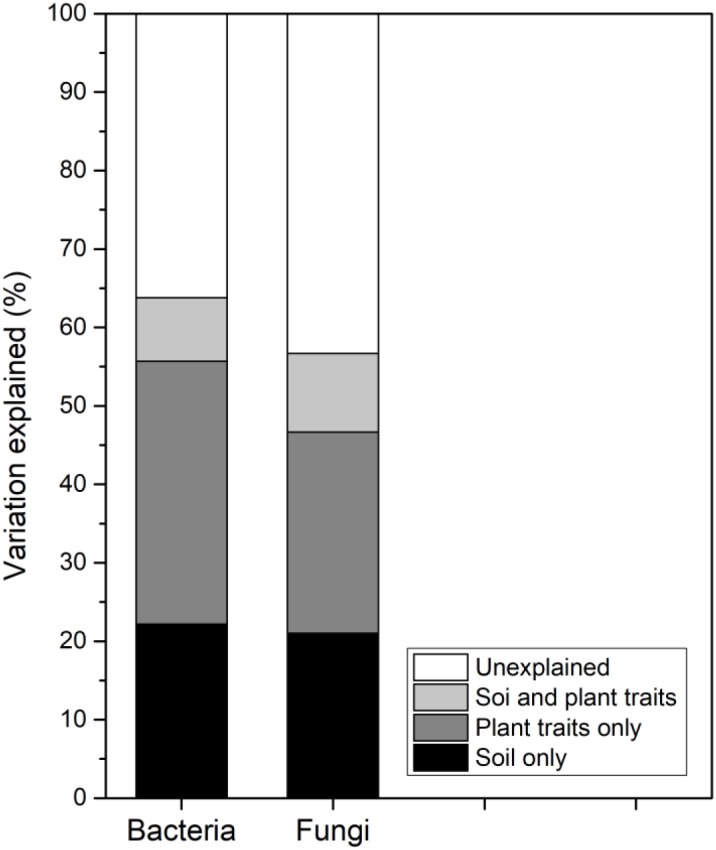
Variation partitioning analysis of the composition of soil bacterial and fungal communities across the six successional stages (S1–S6 represent 1–4, 4–8, 8–15, 15–50, 50–100, and 100–150 years after abandonment, respectively).

Generally, the variations in soil bacterial and fungal community compositions along the successional gradient were strongly associated with the soil abiotic properties and CWM plant traits (Figure 4). The soil properties solely explained 22.17% and 21.03% of the composition of bacterial and fungal communities, respectively. The plant traits solely explained 33.52% and 25.62% of the composition of bacterial and fungal communities, respectively. Further, soil properties and plant traits jointly explain 8.11% and 10.03% of the composition of bacterial and fungal communities, respectively (Figure 5).

All soil abiotic properties (pH, NH_4_-N, NO_3_-N, total N, total C, and PO_4_-P) were significantly associated with the bacterial community composition (Table 2). In addition, soil pH, NH_4_-N, total C, and total N were also significantly associated with the fungal community composition (Table 3). Among these, soil total N was the best predictor of bacterial community composition, while soil pH was the best predictor of fungal community composition. Seven plant traits (height, SLA, SNC, RCC, LPC, SM, and RNC) were significantly associated with the bacterial community composition (Table 2), while five traits (height, leaf N:P, SM, SLA, and RCC) were significantly associated with the fungal community composition (Table 3).

**Table 2:** Significance of the effects of soil abiotic properties and plant functional traits on bacterial community composition along the successional gradient.

Soil property	CCA1	CCA2	*r*^2^	Pr (>*r*)	Significance
**Soil property**					
Total N	0.90	0.43	0.93	0.00	^∗∗∗^
Total C	0.88	0.47	0.87	0.00	^∗∗∗^
NH_4_-N	1.00	-0.07	0.64	0.00	^∗∗∗^
PO_4_-P	0.64	0.77	0.56	0.00	^∗∗∗^
pH	-0.98	0.22	0.41	0.01	^∗∗^
NO_3_-N	-0.91	0.41	0.25	0.03	^∗^
**Plant trait**					
Height	0.86	-0.51	0.79	0.00	^∗∗∗^
SLA	0.25	0.97	0.44	0.00	^∗∗∗^
SNC	-0.42	0.91	0.49	0.00	^∗∗∗^
RCC	0.39	-0.92	0.45	0.00	^∗∗∗^
LPC	-0.32	0.95	0.54	0.00	^∗∗∗^
SM	0.99	-0.11	0.37	0.00	^∗∗^
RNC	-0.04	1.00	0.32	0.01	^∗∗^
LNC	-0.75	0.66	0.20	0.05	NS


*SLA, specific leaf area; SNC, stem nitrogen concentration; RCC, root carbon concentration; LPC, leaf phosphorus concentration; SM, seed mass; RNC, root nitrogen concentration; LNC, leaf nitrogen concentration. NS, no significance, ^∗^*P* < 0.05, ^∗∗^*P* < 0.01, ^∗∗∗^*P* < 0.001.*

**Table 3:** Significance of the effects of soil abiotic properties and plant functional traits on fungal community composition.

Soil property	CCA1	CCA2	*r*^2^	Pr (>*r*)	Significance
**Soil property**					
pH	0.95	-0.30	0.87	0.00	^∗∗∗^
NH_4_-N	-0.93	-0.37	0.53	0.00	^∗∗∗^
Total C	-0.67	0.74	0.43	0.00	^∗∗∗^
Total N	-0.68	0.73	0.29	0.01	^∗∗^
NO_3_-N	0.90	-0.44	0.18	0.11	NS
PO_4_-P	-1.00	-0.02	0.12	0.17	NS
**Plant trait**					
Height	0.90	-0.43	0.88	0.00	^∗∗∗^
L N:P	0.05	-1.00	0.61	0.00	^∗∗∗^
SM	0.73	-0.68	0.49	0.00	^∗∗∗^
SLA	0.36	0.93	0.28	0.01	^∗^
RCC	0.67	-0.74	0.27	0.03	^∗^
SNC	-0.96	0.29	0.15	0.10	NS


*L N:P, leaf N:P ratio; SM, seed mass; SLA, specific leaf area; RCC, root carbon concentration; SNC, stem nitrogen concentration. NS, no significance, ^∗^*P* < 0.05, ^∗∗^*P* < 0.01, ^∗∗∗^*P* < 0.001.*

### Association of Soil Microbial Diversity With Plant Species Diversity and Soil Abiotic Properties

**FIGURE 6:**
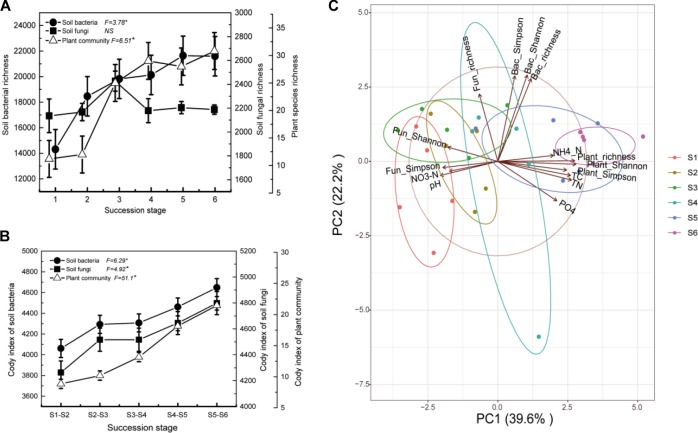
Comparison of plant, bacterial, and fungal community species richness **(A)** and Cody index **(B)** along the successional gradient (S1–S6 represent 1–4, 4–8, 8–15, 15–50, 50–100, and 100–150 years after abandonment, respectively). *F*-values of one-way ANOVA of the effects of succession are provided with the level of significance set at *P* < 0.05. Principal component analysis (PCA) demonstrated the relationships between the soil abiotic properties, plant community diversity, and microbial community diversity **(C)**.

Both bacterial species richness and plant species richness increased significantly along the successional gradient (Figure 6A). The species turnover rate consistently showed an increasing trend from early to later successional stages for the bacterial, fungal, and plant communities (Figure 6B). Further, bacterial alpha diversity was positively correlated with plant species alpha diversity, while fungal diversity was negatively correlated with plant species diversity (Figure 6C). In addition, changes in soil bacterial diversity were strongly correlated with the soil environment; a positive correlation was observed with soil total N, total C, and NH_4_-N, while a negative correlation was observed with soil NO_3_-N and pH. In contrast, soil fungal diversity was positively correlated with soil NO_3_-N and pH, while it was negatively correlated with soil total N, total C, and NH_4_-N (Figure 6C).

The relative abundance of the dominant microbial groups was significantly correlated with the important value index of dominant plant species (Supplementary Figure S9). In the case of fungi, Chytridiomycota, Ascomycota, and Glomeromycota had a positive effect on the important value index of dominant plant species (*Setaria viridis* and *Roegneria kamoji*) at early successional stages, while Basidiomycota had a positive effect on the dominant plant species (*Forsythia suspensa*, *Quercus aliena*, and *Quercus wutaishanica*) at later successional stages. In the case of bacteria, Bacteroidetes had a positive effect on the occurrence of dominant plant species (*S. viridis, Artemisia annua*, and *Cirsium setosum*) at stage 1, while Nitrospirae, Proteobacteria, and Actinobacteria had a positive effect on the dominant plant species (*Prunus davidiana*, *Q. aliena*, and *Q. wutaishanica*) at later successional stages. The other microbial groups were mainly associated with the dominant plant species at the intermediate stages.

### Bacteria–Fungi Interactions Based on Co-occurrence Networks

**FIGURE 7:**
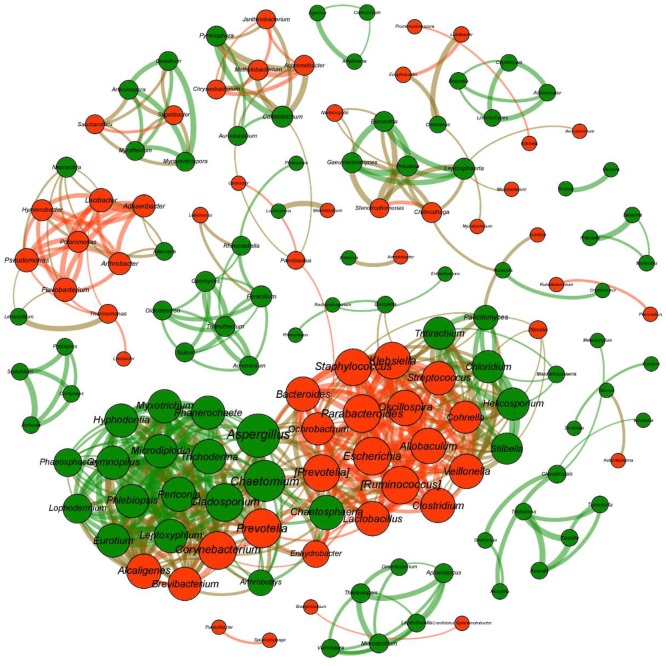
Co-occurrence networks revealing the associations between bacterial taxa and fungal taxa at the genus level. Only strong (| *r*| > 0.8) and significant (*P* < 0.05) Spearman correlations are shown. The number of connections (i.e., degree) is proportional to the size of each node, and the value of Spearman’s correlation coefficients is proportional to the thickness of the connection between two nodes (i.e., edge). The bacterial genera are colored in red, and the fungal genera are colored in green.

To investigate bacterial–fungal interactions, co-occurrence networks were constructed using all bacterial and fungal genera (Figure 7). The network consisted of 147 nodes with the average degree of 7.77, average path length of 2.48, and clustering coefficient of 0.66. The modularity was 0.69, which indicates that the modular structure was evident. Based on the abundance values determined at the genus level, we found a number of significant associations within the bacterial and fungal communities, but few associations were observed between the two communities.

## Discussion

In the present study, we have set out to explore the dynamic patterns of soil bacterial and fungal communities during secondary forest succession, and their associations with plant functional traits and soil abiotic properties over the years of succession on China’s Loess Plateau. Our findings indicate that plant traits could be a link between plant and soil microbial communities while affecting their interactions. Thus, this study provides insight into the mechanisms involved in secondary forest succession in semi-arid ecosystems.

### Changes in Soil Bacterial and Fungal Community Compositions During Secondary Forest Succession

In this study, the soil bacterial communities were mainly composed of Acidobacteria, Proteobacteria, Actinobacteria, Chloroflexi, Planctomycetes, and Bacteroidetes across the six successional stages. This finding is in line with previous studies conducted in the grasslands of the Loess Plateau (Li et al., 2013; Zhang et al., 2016) as well as those in temperate (Shen et al., 2013) and subtropical forest areas (Lin et al., 2012; Delgado-Baquerizo et al., 2018). Although Proteobacteria and Acidobacteria were the most abundant phyla across the six successional stages, we did not find a significant increase in the relative abundance of Proteobacteria along the successional gradient. A similar phenomenon has been reported by other studies that investigated grassland succession (Jangid et al., 2013; Zhang et al., 2016). In the Loess Plateau region, serious water and soil erosion during secondary forest succession may have limited changes in the proportion of Proteobacteria or Acidobacteria, even though these bacteria often play a key functional role in the restoration of soil (Goldfarb et al., 2011). We found that the soil fungal communities were dominated by Ascomycota and Basidiomycota across the six successional stages, and there was a general shift from Ascomycota-dominant to Basidiomycota-dominant communities during the succession. This is consistent with observations of fungal community succession in the forefield of glaciers (Brunner et al., 2011; Dong et al., 2016). Generally, members of the Ascomycota are common in extreme environments, while Basidiomycota members favor resource-rich conditions with high plant species richness (Nara, 2008). Therefore, changes in the fungal communities from Ascomycota-dominant to Basidiomycota-dominant ones indicate the increasing accumulation of soil nutrients and maturing of the ecosystem during the succession.

Our results showed that there was an apparent change in soil bacterial and fungal community compositions during secondary forest succession. PCoA analyses based on UniFrac distances revealed that the soil bacterial communities differed between early (S1-S3) and later successional stages (S4-S6); however, partial overlap between the successional stages was still observed. This is in contrast with the results of Lozano et al. (2014) and Zhang et al. (2016), who reported a clear separation between the successional stages in an abandoned arable field. The reason for the difference might be that we considered not only typical plant communities but also transition types between two adjacent successional stages so as to represent the whole successional process more accurately. Forest community succession involves a long replacement process of the dominant plant species, while the composition of soil microbial communities depends on the identity of plant species and the specific micro-environment at different successional stages (Yeoh et al., 2017). Although the encroachment of later plant species leads to the loss of original taxa and the recruitment of novel lineages, later communities still have some species in common with early communities. Therefore, the differences in soil microbial communities across the six successional stages indicate a gradual increase in vegetation restoration. Specially, although our sample plots were subjected to the same agricultural history, the soil bacterial and fungal communities at the early successional stages still had high heterogeneity. However, the bacterial and fungal communities in the later forest soils (S5 and S6) were more similar to each other compared with those in the early grassland and shrubland soils; thus, the restoration of vegetation could reduce the heterogeneity of microbe assemblages at a local scale.

An indicator species can be regarded as an ecological indicator of environmental changes and community types (Segata et al., 2011), and is effective for predicting the strength of the links between certain habitats and species occurrence (Raiesi and Beheshti, 2015). On the Loess Plateau, each successional stage (exception stage 2) had its unique indicator families for both bacteria and fungi. Moreover, some of these families are either important drivers of particular soil biogeochemical processes or related to certain habitat types. For example, Nitrosomonadaceae, the indicator family for stage 1, is beneficial for improving biogeochemical nitrogen cycling in soils, as is evident in agricultural ecosystems (Cai et al., 2018). Acidobacteriaceae, Hyphomonadaceae, Hyphomicrobiaceae, and Planctomycetaceae, the indicator families for stage 6, may have specialized life history strategies in the soil of the climax forest on the Loess Plateau. These microbial indicators may be useful to further define habitat requirements for aboveground vegetation at each successional stage and better understand their ecological niches. However, further studies are needed to understand the roles of these indicator families in driving secondary forest succession in similar ecosystems.

### Relationships Between Soil Microbial and Plant Community Characteristics During Secondary Forest Succession

A recent study investigated the soil bacterial community dynamics during an herbaceous community’s succession on the Loess Plateau, finding different patterns for plant and bacterial succession (Zhang et al., 2016). By considering a long-term and integrated arable-to-climax forest succession in this plateau region, here we found that both plant species alpha diversity and soil bacterial alpha diversity considerably increased with secondary forest succession. That is, soil bacterial alpha diversity was positively correlated with plant species alpha diversity along the successional gradient. This is because changes in plant diversity from arable to climax forest communities could deeply modify the resource availability and microclimate in soil and thus affect soil bacterial diversity (Zumsteg et al., 2012). However, soil fungal diversity was negatively correlated with plant species diversity; this indicates incongruous changes in soil bacterial and fungal communities in the process of succession. Moreover, the few significant associations observed between bacterial and fungal communities in the present study are also indicative of independent recovery patterns for these two communities. In contrast to our findings, some studies have found that plant diversity is a good predictor of soil fungal diversity in rainforests (Peay et al., 2013), grass land experiments (LeBlanc et al., 2015), and semi-arid sandy grasslands (Zuo et al., 2016). Yet another study on forest ecosystem restoration after mining found that bacterial but not fungal community dynamics followed a pattern in developing ecosystems (Banning et al., 2011). The contrasting dynamics between bacterial and fungal diversity could be explained as follows: (i) bacteria have higher variability between successional stages on account of their higher growth rates (Sun S. et al., 2017), and (ii) the influencing environmental factors during forest succession are different for fungi and bacteria.

Based on the values of the Cody index, we found that the species turnover rate increased along the successional gradient for the bacterial, fungal, and plant communities. This finding underscores the interaction between aboveground plant communities and belowground microbial communities. Similarly, recent studies have also reported that soil microbial community distance patterns at the regional and continental scales were positively associated with aboveground tree assemblage distance patterns in a tropical forest (Barberán et al., 2015) and grasslands worldwide (Prober et al., 2015). At the genus level, we found that the relative abundance of the dominant bacterial and fungal genera was distinct across successional stages, while closely related genera appeared to dominate at certain successional stages. Further analysis identified the relationships between the important value index of dominant plant species and the relative abundance of the dominant microbial groups. We found that certain fungal and bacterial taxa tended to be strongly associated with the occurrence of dominant plant species. For example, the fungal phyla Chytridiomycota, Ascomycota, and Glomeromycota showed a positive effect on the important value index of *S. viridis* and *R. kamoji* at early successional stages, while Basidiomycota promoted the occurrence of *F. suspensa*, *Q. aliena*, and *Q. wutaishanica* at later successional stages. Previous studies have confirmed that the identity of a plant species determines the type of soil microbes found in the rhizosphere (Tscherko et al., 2004; Peiffer et al., 2013). Here our results indicate that the soil microbial community affected plant community assembly and could be partially responsible for the replacement of dominant plant species during secondary forest succession in semi-arid ecosystems.

### Contributions of Soil Abiotic Properties and CWM Plant Traits to Microbial Community Composition During Secondary Forest Succession

Recent studies have clarified the roles of soil properties and plant traits in explaining variations in soil microbial community composition at the regional and landscape scales (de Vries et al., 2012; Legay et al., 2014; Sterkenburg et al., 2015). Here, we found that the composition and diversity of soil bacterial and fungal communities were correlated with both soil abiotic properties and CWM plant traits along the secondary forest successional gradient. Thus, it seems that both soil abiotic properties and CWM plant traits are significant drivers of soil microbial community composition during secondary forest succession.

In this study, the soil properties significantly explained the composition of both the bacterial and fungal communities. In this regard, some studies have confirmed the effects of soil pH on bacterial or fungal community compositions at the local scale (Rousk et al., 2010), regional scale (Siciliano et al., 2014; Tripathi et al., 2015), and continental scale (Fierer et al., 2009; Griffiths et al., 2011). Here, we found that soil total N and pH were, respectively, the best predictors of soil bacterial and fungal community compositions during secondary forest succession on the Loess Plateau. This difference in the soil indicators may explain why the bacterial and fungal communities showed independent recovery patterns during succession. On the one hand, smaller scale studies have suggested that bacterial communities are more responsive to pH changes than fungal communities (Rousk et al., 2010). Generally, bacterial diversity peaks at intermediate pH in terrestrial ecosystems (Fierer and Jackson, 2006). In the present study, although soil pH declined along the successional gradient on the Loess Plateau, the soil pH of later successional stages was close to neutral (pH, ∼7). This may explain the higher bacterial diversity observed at later successional stages. However, nitrogen is widely acknowledged as being an important limiting nutrient factor on the Loess Plateau (Chai et al., 2016); thus, the nitrogen concentration might still be a major regulator of the bacterial community composition. The strong dependence of bacterial communities on soil nitrogen illustrates that nitrogen dynamics may be more important than pH in determining bacterial responses to vegetation restoration during forest succession. On the other hand, the greater influence of soil pH on fungal community composition than on bacterial community composition observed in the present study is probably attributable to the intensive interaction between pH and other factors, as fungi are generally able to tolerate a wider range of pH than bacteria (Rousk et al., 2010). Rousk et al. (2010) argued that the observed pattern between the fungal community composition and soil pH is an indirect effect that is mediated by the competitiveness and strong dynamics of the bacterial community along the pH gradient. Nonetheless, the fungal community composition does seem to be sensitive to soil pH in semi-arid ecosystems. The effects of soil pH on fungal diversity we observed are also in line with the results of a study that were conducted in other vegetation types such as grasslands (Yang et al., 2014).

Community-weighted mean is defined as the abundance-weighted mean trait value for a plant community (Diaz et al., 2007) that reflects the resource-use strategy of plants (Conti and Diaz, 2013). Considerable evidence has confirmed the effects of CWM plant traits on the community assembly and ecosystem process (Butterfield and Suding, 2013; Conti and Diaz, 2013). In the present study, the community-level leaf/stem/root carbon concentration and dry matter content increased with the successional gradient: that is, later dominant plants showed a tendency toward more conservative resource use strategies (Milcu et al., 2014), while exploitative species with higher SLA and leaf/stem/root nitrogen content were dominant at early successional stages. CCA analyses showed that plant traits explained approximately one-third of the variation in bacterial and fungal community compositions. Some studies have also found that the plant functional traits related to growth rate and resource utilization strategies regulate bacterial community composition via mediation of soil carbon, nitrogen, and phosphorus cycling (Orwin et al., 2010; Chu et al., 2011; de Vries et al., 2012; Ke et al., 2015). For example, at the species level, fast-growing exploitative species select for bacteria-dominated communities in grasslands (Orwin et al., 2010). Our results further showed that plant height, SM, LPC, SLA, SNC, and RNC were also significantly related to the composition of soil bacterial and fungal communities during secondary forest succession on the Loess Plateau. However, other studies have found that plant traits are not effective indicators of the plot-level variability in belowground microbial communities (Barberán et al., 2015; Leff et al., 2018). It is possible that our results differed because we used the abundance-weighted mean trait value for a community, which reflects the resource-use strategy of the dominant plant species (which mainly reflects carbon and nitrogen cycling at the community level). Another possible reason is that the plant traits changed more rapidly during secondary forest succession on the Loess Plateau than in other ecosystems, thus enhancing the strength of the interactions between the plant and soil microbial communities.

Collectively, the results described so far indirectly support the relevance of the soil microbial community not only to soil processes, but also to aboveground bioprocesses, as per a previously proposed hypotheses (De Deyn et al., 2008; Laughlin, 2011). However, variation partitioning revealed that 36.20% and 43.32% of the total variance in bacterial and fungal community compositions, respectively, could not be explained by soil properties and plant traits; this means that other factors also contribute to the variation in soil microbial diversity. Thus, some unmeasured traits, such as litter carbon chemistry or other root traits (Cantarel et al., 2015), may be important drivers of soil microbial community composition during secondary forest succession.

## Conclusion

In this study, we found substantial changes in soil bacterial and fungal community compositions during a long-term secondary forest succession on the Loess Plateau. The soil bacterial and fungal communities showed distinct response patterns to plant community succession. Our results highlighted the strong dependence of soil microbial community composition and diversity not only on the soil abiotic properties, but also on the plant functional traits related to resource acquisition and utilization. These results indicate that plant traits could be a link between plant and soil microbial communities and affect their interactions during secondary forest succession. These findings improve our understanding of the succession mechanisms. In the future, more studies are required to quantify these indirect relationships between soil microbial properties and plant community dynamics that are mediated by plant traits.

## Author Contributions

YoC, YiC, and MY designed the experiments and wrote the manuscript. TT, QY, HD, JQ, RZ, and MW provided critical help in the field work and with data analysis.

## Conflict of Interest Statement

The authors declare that the research was conducted in the absence of any commercial or financial relationships that could be construed as a potential conflict of interest.
