# Supplementary material for: Soil Abiotic Properties and Plant Functional Traits Mediate Associations Between Soil Microbial and Plant Communities During a Secondary Forest Succession on the Loess Plateau
Source: Front Microbiol. 2019 Apr 26;10:895. doi: 10.3389/fmicb.2019.00895 (PMC6499021; doi:10.3389/fmicb.2019.00895)
Supplement: Supplementary file 1 [file Table_1.docx]

**SUPPLEMENTARY MATERIAL**

**TABLE S1 |** Main characteristics of the vegetation community across the six successional stages on the Loess Plateau.

| **Successional stage** | **Vegetation community** | **Slope aspect** | **Slope gradient** | **Coverage (%)** |
| --- | --- | --- | --- | --- |
| S1 (1–4 years) | *Setaria viridis*  *Artemisia annua*  *Cirsium setosum* | SE10° | 18°–20° | 20–30% |
| S2 (4–8 years) | *Artemisia gmelinii*  *Artemisia subdigitata*  *Artemisia lavandulaefolia* | SE15° | 20° | 35–40% |
| S3 (8–15 years) | *Miscanthus sacchariflorus*  *Bothriochloa ischaemum*  *Roegneria kamoji*  *Poa sphondylodes* | SE15° | 18°-20° | 50–60% |
| S4 (15–50 years) | *Ostryopsis davidiana*  *Sophora davidii*  *Forsythia suspensa*  *Spiraea trilobata*  *Rosa hugonis* | S | 20° | 65–75% |
| S5 (50–100 years) | *Pinus tabulaeformis*  *Platycladus orientalis*  *Prunus davidiana* | S | 23°-25° | 75–85% |
| S6 (100–150 years) | *Quercus aliena*  *Quercus wutaishanica* | S | 23° | 75–80% |

**TABLE S2 |** List of the 15 plant functional traits, their ecological strategies, and six soil abiotic properties tested in this study.

| **Group** | **Trait** | **Abbreviation** | **Unit** | **Strategy** |  |
| --- | --- | --- | --- | --- | --- |
| Leaf | Leaf nitrogen content | LNC | mg/g | Resource capture |  |
|  | Leaf carbon content | LCC | mg/g | Resource capture and defense |  |
|  | Leaf phosphorus content | LPC | mg/g | Resource capture |  |
|  | Leaf N:P ratio | N:P | ratio | Resource capture |  |
|  | Leaf dry mass content | LDMC | mg/g | Leaf structure |  |
|  | Specific leaf area | SLA | m^2^/kg | Resource capture |  |
| Stem | Stem nitrogen content | SNC | mg/g | Resource capture |  |
|  | Stem carbon content | SCC | mg/g | Resource capture and defense |  |
|  | Stem specific density | SSD | g/cm^3^ | Stem structure |  |
|  | Stem dry mass content | SDMC | mg/g | Stem structure |  |
| Root | Root nitrogen content | RNC | mg/g | Resource capture |  |
|  | Root carbon content | RCC | mg/g | Resource capture and defense |  |
|  | Root dry mass content | RDMC | mg/g | Root structure |  |
| Seed | Seed mass | SM | g per 1000 seeds | Dispersal ability |  |
| Plant | Plant height | Height | m | Light competition |  |
| Soil | pH, Total C, Total N, NO_3_-N, NH_4_-N, and PO_4_-P | | | |  |

**TABLE S3 |** Summary of soil microbial community composition and alpha diversity indices by successional stage.

| **Bacterial community characteristics** | | **Successional stage** | | | | | | | | | |
| --- | --- | --- | --- | --- | --- | --- | --- | --- | --- | --- | --- |
|  |  | **S1** | | **S2** | | **S3** | | **S4** | | **S5** | **S6** |
| Number of genera | 289 | | 288 | | 284 | | 278 | | 272 | | 247 |
| Number of families | 228 | | 218 | | 218 | | 223 | | 206 | | 204 |
| Number of orders | 187 | | 187 | | 180 | | 182 | | 177 | | 183 |
| Number of classes | 120 | | 124 | | 121 | | 114 | | 117 | | 120 |
| Number of phyla | 42 | | 44 | | 44 | | 41 | | 41 | | 40 |
| Simpson diversity | 0.96±0.00 | | 0.98±0.00 | | 1.00±0.00 | | 0.99±0.01 | | 1.00±0.00 | | 1.00±0.00 |
| Shannon diversity | 10.02±0.47 | | 10.33±0.28 | | 10.63±0.16 | | 10.17±0.87 | | 10.65±0.32 | | 10.71±0.30 |
| Chao1 index | 14979.18±4964.51 | | 18455.12±4982.73 | | 19823.04±1756.28 | | 20128.66±2007.49 | | 21633.93±2134.25 | | 21591.49±1601.67 |
| Observed species | 5282.20±1174.59 | | 6091.20±947.35 | | 6623.60±409.23 | | 5870.60±1701.85 | | 6817.00±576.21 | | 6927.00±435.66 |
| **Fungal community characteristics** | **S1** | | **S2** | | **S3** | | **S4** | | **S5** | | **S6** |
| Number of genera | 264 | | 250 | | 282 | | 268 | | 278 | | 272 |
| Number of families | 131 | | 130 | | 140 | | 138 | | 139 | | 138 |
| Number of orders | 61 | | 60 | | 64 | | 62 | | 61 | | 61 |
| Number of classes | 20 | | 20 | | 22 | | 21 | | 19 | | 20 |
| Number of phyla | 5 | | 5 | | 5 | | 5 | | 5 | | 5 |
| Simpson diversity | 0.93±0.03 | | 0.89±0.10 | | 0.92±0.06 | | 0.89±0.08 | | 0.87±0.07 | | 0.82±0.08 |
| Shannon diversity | 5.76±1.06 | | 5.56±1.33 | | 6.15±1.10 | | 5.54±1.08 | | 5.36±0.62 | | 4.82±0.97 |
| Chao1 index | 2135.71±563.34 | | 2167.31±288.45 | | 2426.22±335.64 | | 2179.29±402.71 | | 2203.98±205.50 | | 2187.77±157.46 |
| Observed species | 1362.40±413.57 | | 1420.80±277.72 | | 1632.60±189.50 | | 1446.00±309.99 | | 1474.60±155.55 | | 1436.00±161.79 |

**TABLE S4**

Effect of plant community succession on soil microbial community composition by Adonis and Anosim tests.

| Between groups | | Bacteria | | Fungi | |
| --- | --- | --- | --- | --- | --- |
|  |  | Adonis | Anosim | Adonis | Anosim |
| S1 | S4 | 0.56 | 0.71 | 0.46 | 0.42 |
| S1 | S5 | **0.01** | **0.01** | **0.03** | **0.04** |
| S1 | S6 | **0.01** | **0.01** | **0.01** | **0.01** |
| S2 | S1 | 0.67 | 0.52 | 0.74 | 0.80 |
| S2 | S4 | 0.67 | 0.75 | 0.86 | 0.76 |
| S2 | S5 | **0.04** | **0.03** | 0.24 | 0.17 |
| S2 | S6 | **0.03** | **0.04** | **0.11** | **0.06** |
| S3 | S1 | 0.82 | 0.64 | 0.42 | 0.22 |
| S3 | S2 | 0.99 | 0.91 | 0.75 | 0.36 |
| S3 | S4 | 0.51 | 0.54 | 0.42 | 0.20 |
| S3 | S5 | **0.01** | **0.03** | **0.04** | **0.03** |
| S3 | S6 | **0.01** | **0.01** | **0.01** | **0.01** |
| S5 | S4 | 0.08 | 0.08 | 0.83 | 0.85 |
| S6 | S4 | **0.02** | **0.04** | 0.67 | 0.38 |
| S6 | S5 | 0.98 | 0.89 | 0.88 | 0.77 |

Bold values indicate significant effects (*P* < 0.05).

**

** **FIGURE S1.** Relative abundance of the soil bacterial (a) and fungal (b) communities at the phylum level across the six susccetional stages (S1 to S6 represent 1–4, 4–8, 8–15, 15–50, 50–100, and 100–150 years after abandonment, respectively). Values are mean±standard error (*n* = 5). Different letters indicate significant differences (*P* < 0.05) across the successional stages.


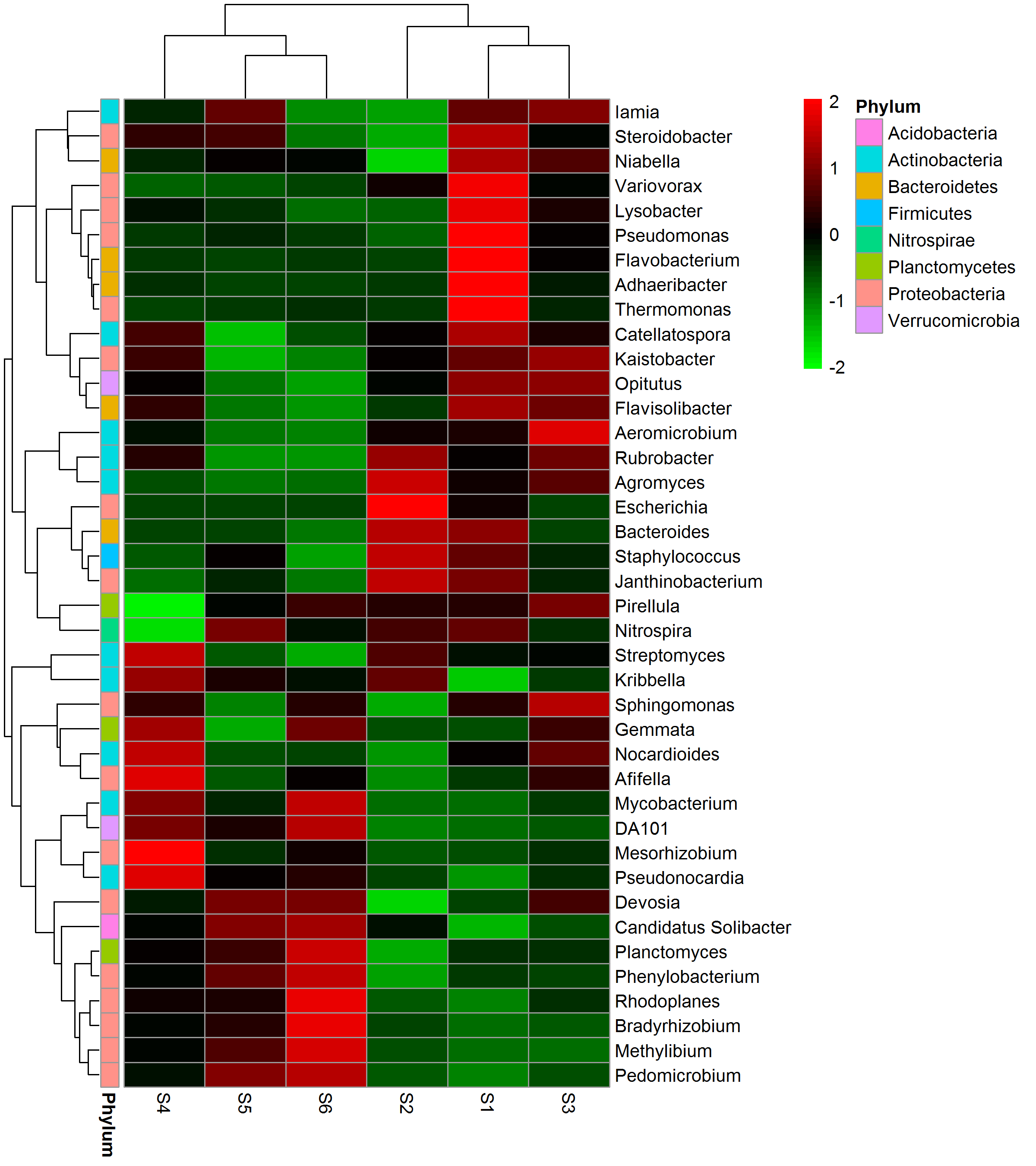


**FIGURE S2.** Bacterial distribution at the genus level across the six successional stages (S1 to S6 represent 1–4, 4–8, 8–15, 15–50, 50–100, and 100–150 years after abandonment, respectively). The heatmap was constructed by R package "pheatmap" and only the top 40 dominant genera were shown. Sample or tax clustering was applied by hclust in complete method. The color bar stands for the relative abundance of each genus in the soil samples from specific successional stage.

**
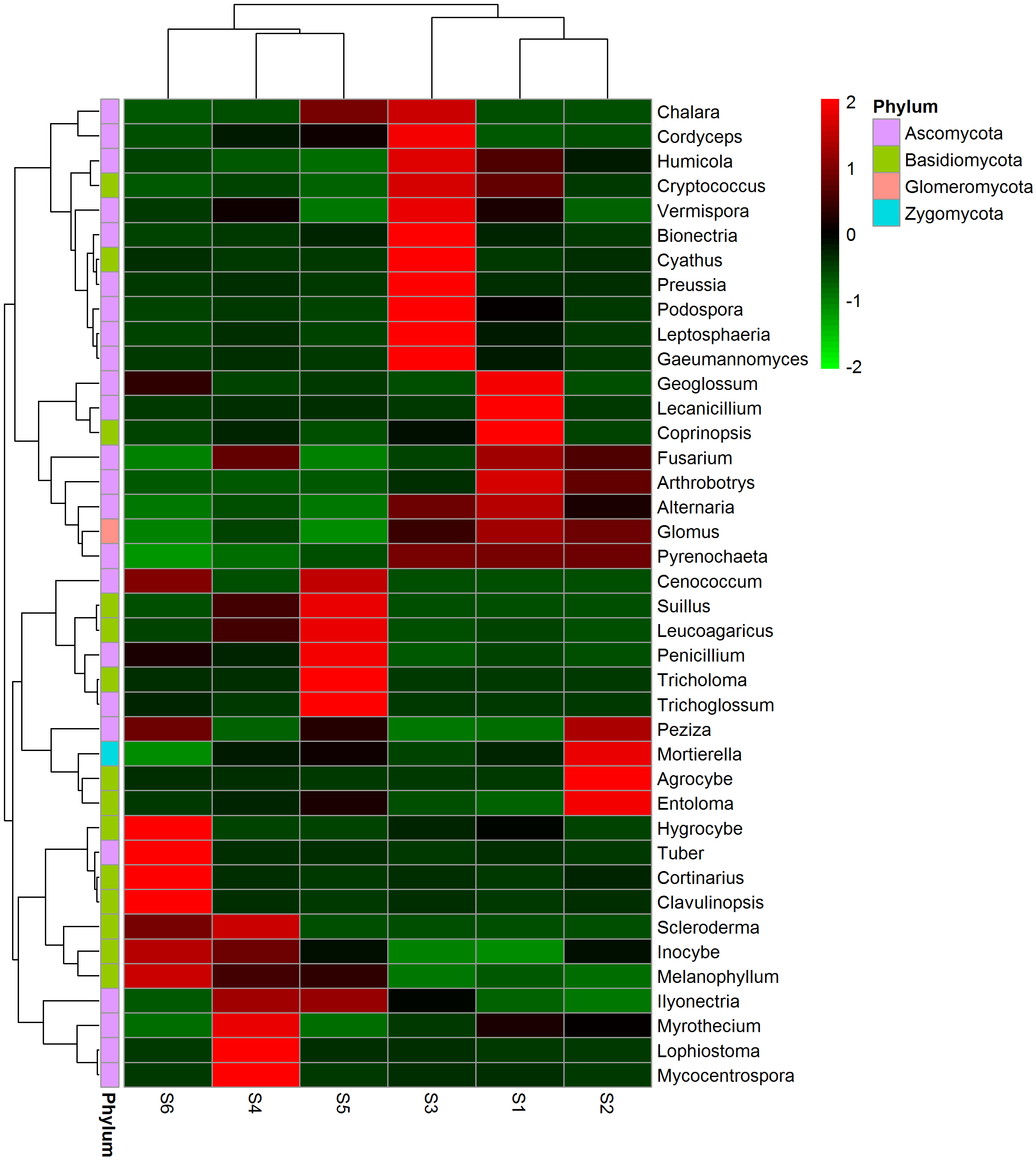
**

**FIGURE S3.** Fungal distribution at the genus level across the six successional stages (S1 to S6 represent 1–4, 4–8, 8–15, 15–50, 50–100, and 100–150 years after abandonment, respectively). The heatmap was constructed by R package "pheatmap" and only the top 40 dominant genera were shown. Sample or tax clustering was applied by hclust in complete method. The color bar stands for the relative abundance of each genus in the soil samples from specific successional stage.


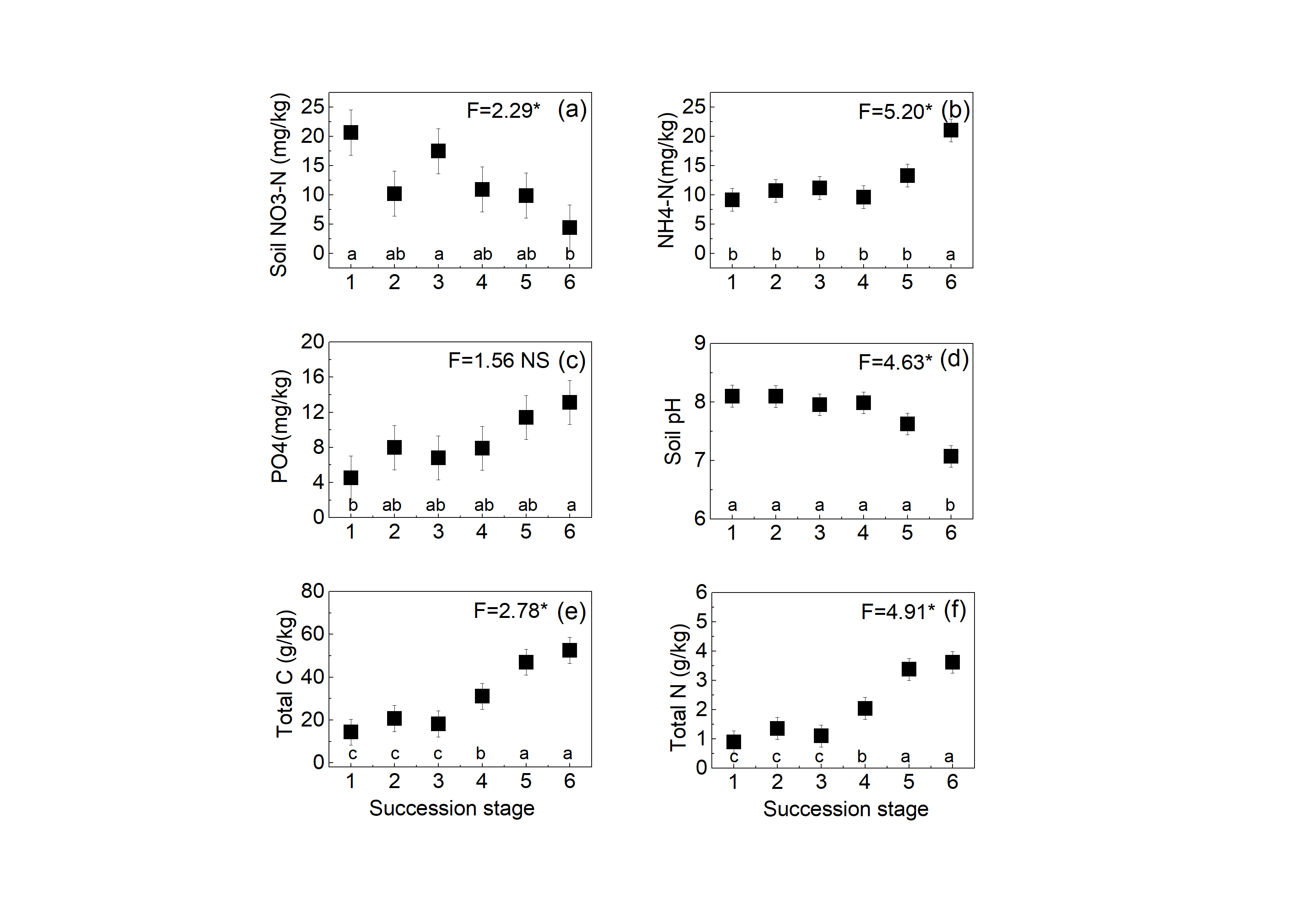


**FIGURE S4.** Patterns of variations in soil abiotic properties along the successional gradient. **(a)** NO_3_-N concentration, (**b)** NH_4_-N concentration, (**c**) PO_4_-P concentration, (**d**) pH level, (**e**) total C concentration, and (**f**) total N concentration. F-values of one-way analysis of variance are given with level of signiﬁcance (* *P* < 0.05). Different letters correspond to results of post-hoc tests. NS represents no significant difference across the successional stages.

**FIGURE S5.** Patterns of variations in community-weighted mean values of leaf traits along the successional gradient. (**a**) Speciﬁc leaf area (SLA), (**b**) leaf dry mass content (LDMC), (**c**) leaf nitrogen content (LNC), (**d**) leaf carbon content (LCC), (**e**) leaf phosphorus content (LPC),and (**f**) leaf N:P ratio (N:P ratio). F-values of one-way analysis of variance are given with level of signiﬁcance (* *P* < 0.05). Different letters correspond to results of post-hoc tests. NS represents no significant difference across the successional stages.


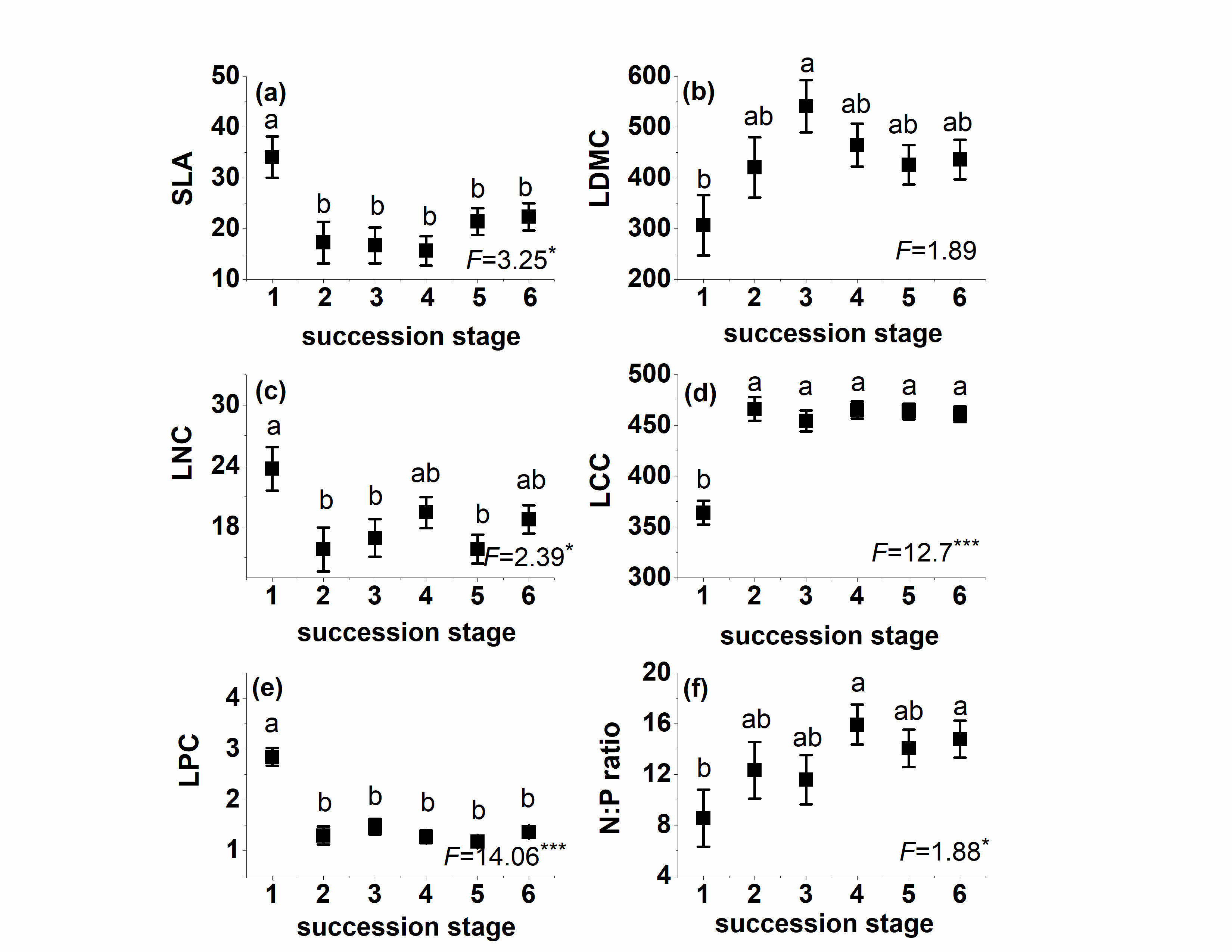

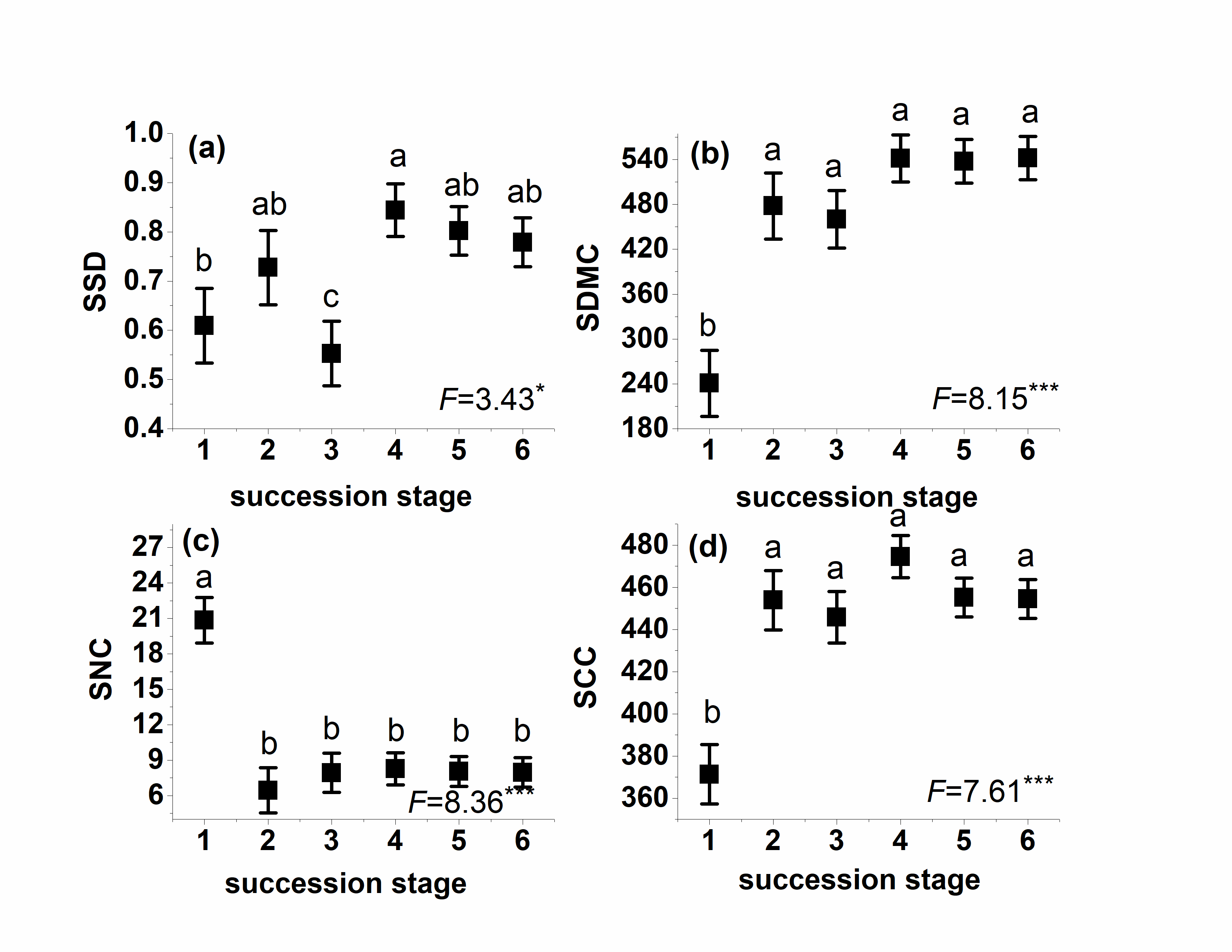


**FIGURE S6.** Patterns of variations in community-weighted mean values of stem traits along the successional gradient. (**a**) Stem specific density (SSD), (**b**) stem dry mass content (SDMC), (**c**) stem nitrogen content (SNC), and (**d**) stem carbon content (SCC). F-values of one-way analysis of variance are given with level of signiﬁcance (* *P* < 0.05). Different letters correspond to results of post-hoc tests. NS represents no significant difference across the successional stages.


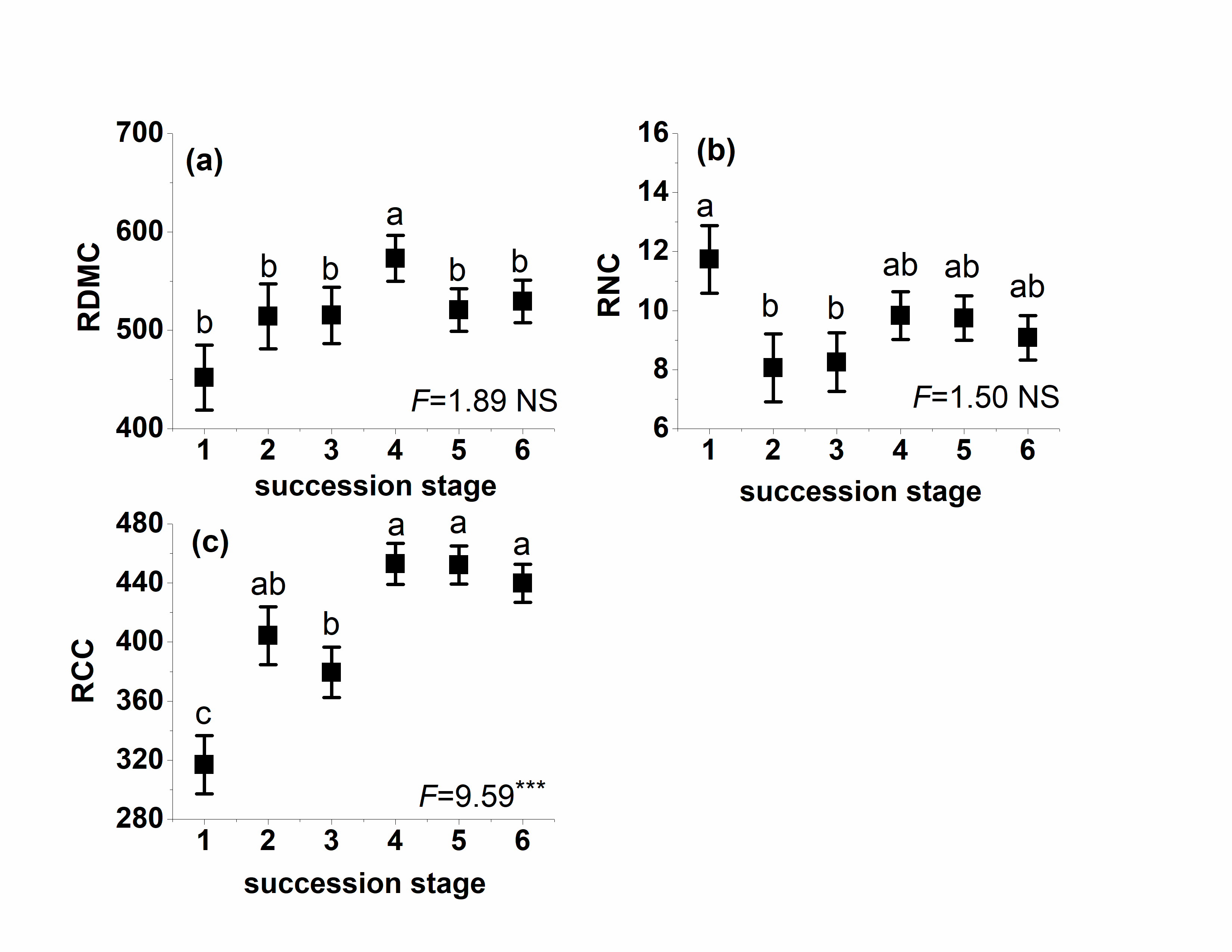


**FIGURE S7.** Patterns of variations in community-weighted mean values of root traits along the successional gradient. (**a**) root dry mass content (RDMC), (**b**) root nitrogen content (RNC), (**c**) root carbon content (RCC). F-values of one-way analysis of variance are given with level of signiﬁcance (* *P* < 0.05). Different letters correspond to results of post-hoc tests. NS represents no significant difference across the successional stages.


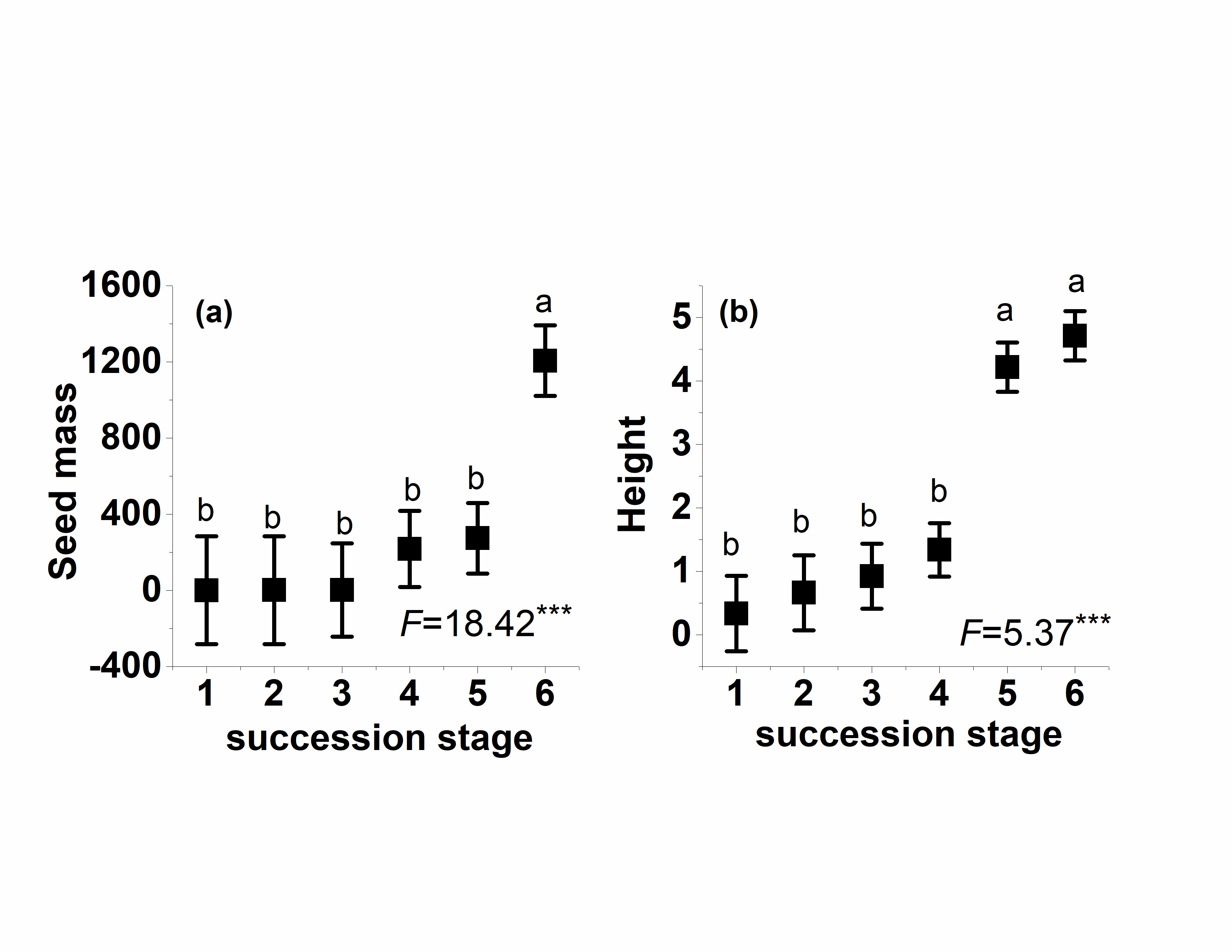


**FIGURE S8.** Patterns of variations in community-weighted mean values of leaf traits along the successional gradient. (a) Seed mass and (b) plant Height. F-values of one-way analysis of variance are given with level of signiﬁcance (* *P* < 0.05). Different letters correspond to results of post-hoc tests. NS represents no significant difference across the successional stages.


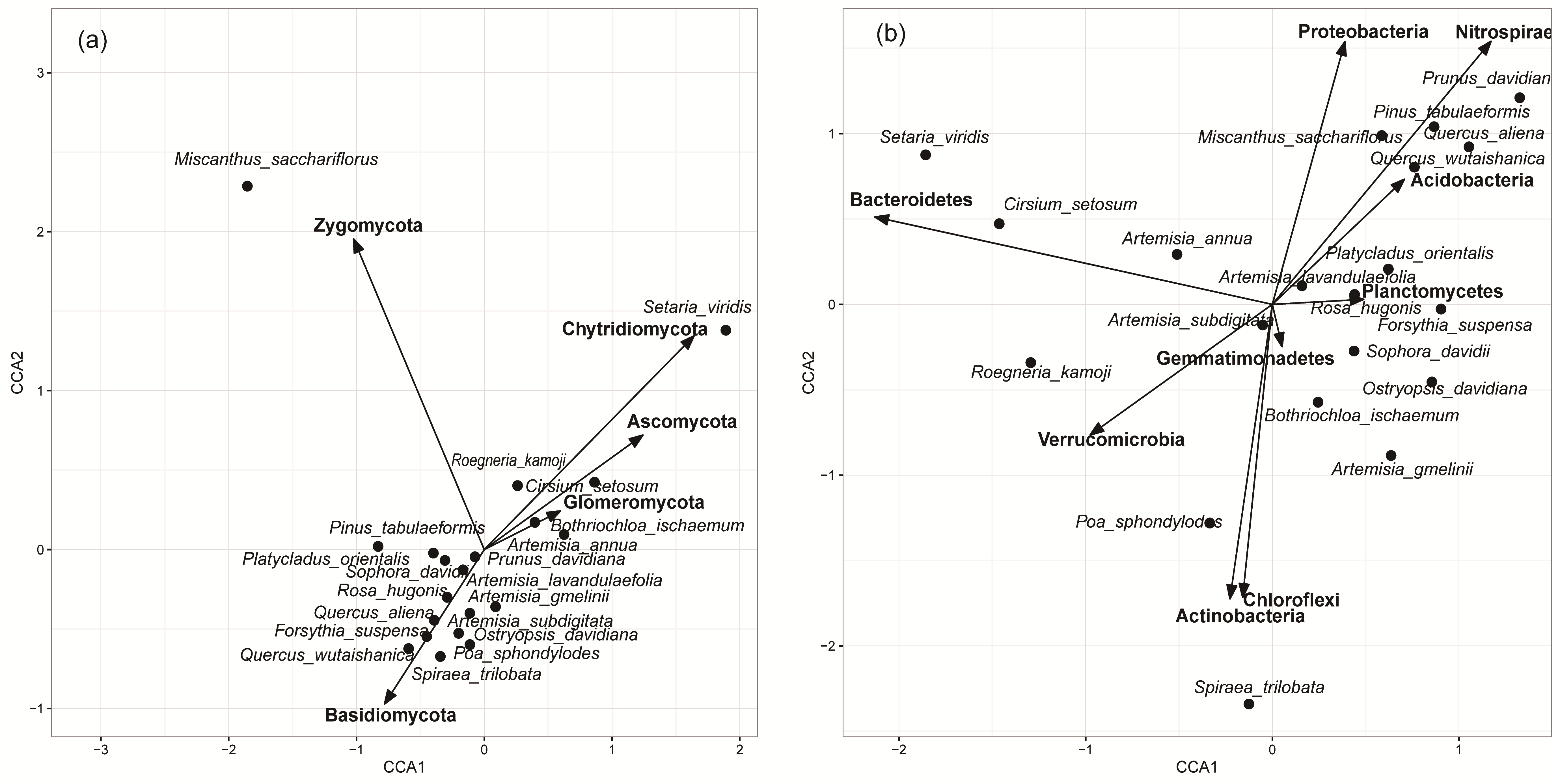


**FIGURE S9.** Canonical correspondence analysis (CCA) showing relationships between the important value of dominant plant species and the relative abundance of the dominant fungal (**a**) and bacterial groups (**b**) at the phylum level. F-values of Monte Carlo permutation testing are significant at *P* < 0.05.
